# Cortical GABAergic Interneurons in Cross-Modal Plasticity following Early Blindness

**DOI:** 10.1155/2012/590725

**Published:** 2012-06-07

**Authors:** Sébastien Desgent, Maurice Ptito

**Affiliations:** ^1^Centre de Recherche du Centre Hospitalier Universitaire (CHU) Sainte-Justine, Université de Montréal, Case Postale 6128, succursale Centre-Ville, Montréal, QC, Canada H3C 3J7; ^2^Département de Physiologie, Université de Montréal, Case Postale 6128, Succursale Centre-Ville, Montréal, QC, Canada H3C 3J7; ^3^Harland Sanders Chair in Visual Science, École d'optométrie, Université de Montréal, Case Postale 6128, succursale Centre-Ville, Montréal, QC, Canada H3C 3J7; ^4^Institute of Neuroscience and Pharmacology and Panum Institute, University of Copenhagen, 2200 Copenhagen, Denmark

## Abstract

Early loss of a given sensory input in mammals causes anatomical and functional modifications in the brain via a process called cross-modal plasticity. In the past four decades, several animal models have illuminated our understanding of the biological substrates involved in cross-modal plasticity. Progressively, studies are now starting to emphasise on cell-specific mechanisms that may be responsible for this intermodal sensory plasticity. Inhibitory interneurons expressing *γ*-aminobutyric acid (GABA) play an important role in maintaining the appropriate dynamic range of cortical excitation, in critical periods of developmental plasticity, in receptive field refinement, and in treatment of sensory information reaching the cerebral cortex. The diverse interneuron population is very sensitive to sensory experience during development. GABAergic neurons are therefore well suited to act as a gate for mediating cross-modal plasticity. This paper attempts to highlight the links between early sensory deprivation, cortical GABAergic interneuron alterations, and cross-modal plasticity, discuss its implications, and further provide insights for future research in the field.

## 1. Introduction

**Figure 1:**
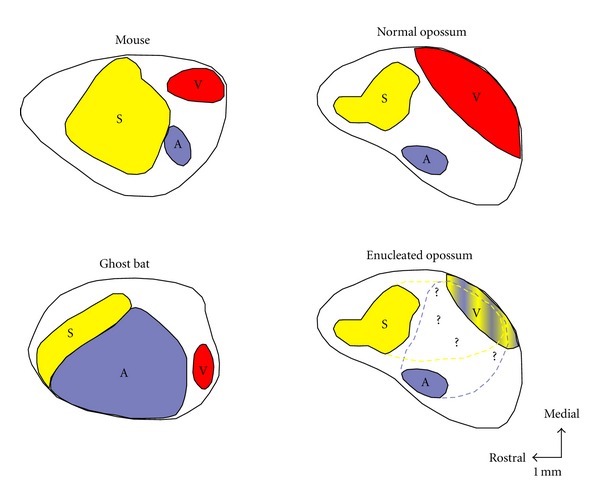
Primary cortical areas in three species of mammals (i.e., Mouse, Ghost Bat and Opossum) that have approximately the same size cortical sheet, but different amounts of cortex allowed to different sensory modality (S: Somatosensory system, A: Auditory system and V: Visual system), related to use of particular sensory receptor arrays. In the mouse (top left), which relies heavily on tactile inputs from the whiskers for survival, the somatosensory cortex (S) is enlarged, compared with the ghost bat (bottom left) and normal opossum (top right). The auditory cortex (A) in the neocortex of the echolocating ghost bat is expanded, while the visual area (V) and S is relatively small. Similarly, the cortex of the highly visual opossum have a dominant visual cortex. Finally, for example, in the enucleated at birth opossum (bottom right) the V cortex becomes smaller and is recruited by the A and S modalities. Similarity in relative location of sensory cortical fields in all these mammals suggests that the topographic organization and overall pattern of thalamocortical projections of the brain is constrained by developmental mechanisms. Conversely, the differences in size, shape, and detailed organization of sensory cortical fields indicate that input from the periphery is a crucial factor in guiding many of the details of organization of the neocortex. Rostral is to the left and medial is up. Scale bar = 1 mm. Adapted from Kahn and Krubitzer, 2002 [48].

Patterns of activity from the peripheral sensory receptor arrays can dramatically influence the development of connectivity and functional organization of cortical fields in mammals. In some species, evolution in relation to specific environmental cues has nurtured the brain's blueprint in such a way that a sensory cortex processing specific survival needs has been enlarged over time as compared to other modalities (Figure 1) [1–5]. Similarly, when a sensory function is lost during development, spared senses compensate by taking more cortical space and recruiting the deafferented areas, to maintain homeostasis of sensory function. This reorganization optimizes and secures the individual's survival and awareness to future environmental changes. For example, the loss of sight at birth or during early life in humans leads to important anatomical and functional reorganization of the visually deprived cortex that will become activated by a wide variety of nonvisual stimuli involving touch, audition, and olfaction [6–11]. Enhanced spatiotemporal functions in the remaining sensory modalities have also been reported [12–16]. It seems therefore that the visual cortex of the blind is not lifeless and is capable of adapting in order to accommodate these nonvisual inputs through cross-modal plasticity. 

But how does a visually deprived cortical area signal its loss of sensory inputs to, or be recruited by, areas of other sensory modalities? Two main hypotheses have been proposed to explain cross-modal plasticity in a visually deprived brain. The first hypothesis proposes that early deprived visual cortical circuits can be rewired and/or cross-wired with other modalities following the initial insult [17–19]. This rewiring stipulates the formation of new and permanent aberrant connections from the sensory receptors of spared modalities to visual thalamic relays and up into the visual cortices. The second hypothesis stipulates the activation, formation, and/or enhancement of corticocortical connections that involve local connectivity modifications in the deprived cortex as well as physically present but functionally silent connections between sensory cortices that could therefore be activated and/or sprout following a specific sensory loss. Thus, early blindness could lead either to abnormal thalamocortical or corticocortical connections. These connections are not yet fully understood but would explain, in part, how the afferents of the remaining modalities could reach the deprived cortex. In order to clarify these hypotheses at the microscale level and to better understand the biological underpinnings of cross-modal plasticity, several early developmental models have been developed in the past four decades. 

Even if it is now widely accepted that cross-modal plasticity involves important anatomical and functional changes in the neocortex, its cellular mechanisms are still ill-defined. Inhibitory GABAergic interneurons are believed to subserve cross-modal plasticity processes such as in re-establishing homeostasis when the excitation-inhibition balance is perturbed. For example, GABAergic neuronal activity coordinates the rhythmic behavior of principal (excitatory) neurons in the cortical networks. GABAergic neurons are also critically involved in neuronal growth, fine tuning of sensory receptive fields, visual plasticity, and the formation of critical periods in development. In addition, GABAergic interneurons especially those expressing calcium-binding proteins like calbindin (CB), calretinin (CR), and particularly parvalbumin (PV) have a protracted development reaching their neurochemical and innervation maturity only during early postnatal life making them very sensitive to sensory experience, sensory privation, and noxious environmental changes. Finally, GABAergic interneurons play a pivotal role in gating sensory thalamocortical feed-forward inputs [20–22], cortico-cortical [23–25], and corticothalamocortical connectivities between visual cortices [26] which is of prime interest for cross-modal plasticity following an early sensory loss. Further, significant morphological alterations in inhibitory networks are found in animal models of sensory deprivation, early blindness, rewiring, and cross-modal plasticity [1, 27–30]. Since interneurons play such a significant role in activity-dependent modification of developing sensory circuits, it is thus important to study specific implications of the various GABAergic subpopulations in cross-modal plasticity paradigms.

In this paper we will first discuss general anatomical and functional findings in different animal models of cross-modal plasticity (in particular hamsters, ferrets, opossum, and mice) and the effects of loss of sensory function on GABAergic cortical networks. We will then focus on how aberrations in inhibitory circuitry could explain cross-modal plasticity and briefly discuss future research directions in the field. Thus, the main objective of this paper is to stress out the importance of studying the GABAergic networks in animal models of cross-modal plasticity for future experimental work because information on the possible mechanisms involved is presently lacking. 

## 2. Animal Models of Cross-Modal Plasticity

 During development, activity pattern amongst different sensory modalities determines the relative size and organization of its representative subcortical and cortical areas. The loss or decrease of any one modality leads to the invasion of the deprived cortical area by inputs originating from other modalities, illustrating the remarkable capacity of the cerebral cortex for plasticity resulting in anatomical reorganization, functional and behavioral recovery. As mentioned before, in blindness, cross-modal changes most probably require or involve rewiring and cross-wiring of cortices. The remaining modalities could colonize the deprived visual cortex directly through changes at the subcortical level (modified thalamo-cortical afferences) and via cortico-cortical connections.

### 2.1. Early Sensory Privation, Binocular Enucleation, and Congenital Blindness Models

 Several studies have shown that the total or partial loss of a sensory modality like vision leads to changes in the anatomical and functional organization of the structures associated with the affected sensory input as well as from the spared modalities [31, 32]. In the 1970s, Rebillard et al. reported for the first time that the primary auditory cortex in congenitally deaf cats could be driven by visual or somatosensory stimuli [33] and was later shown also in a congenitally deaf mouse strain [34]. Furthermore, following bilateral lid sutures at birth in kittens it was also found that the neurons in the visual part of the anterior ectosylvian cortex (AEV) could respond to other modalities [35, 36]. Thus, areas normally dedicated to vision could be taken over by neighbouring auditory and somatosensory areas leading to superior performance in localization discrimination tasks relying on these remaining senses. These changes were attributed to the expansion of the auditory and somatosensory areas to the detriment of extrastriate or associative visual cortices [37, 38]. Cross-modal plasticity has also been observed in primary sensory cortices. In rats enucleated at birth, the primary somatosensory cortex (S1) can recruit the rostral part of primary visual cortex (V1) conferring functional tactile neuronal responses in that area. These rats showed better exploring skills and higher whisker responsiveness than control siblings [39, 40]. Similar anatomical and electrophysiological findings have been reported in early postnatal and adult enucleated mice and rabbits [41–43]. Enucleation at birth or congenital microphthalmia in kittens induces auditory activation of the visual cortex, principally area V1 [44, 45], which has also been shown in hamsters, opossums, and mice [46–49].

**Figure 2:**
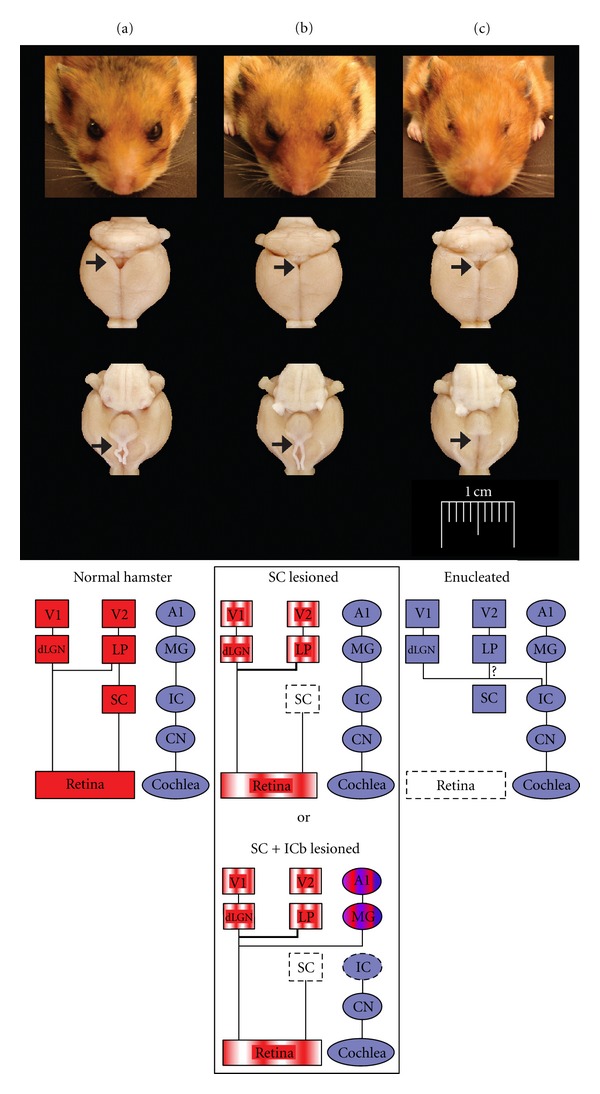
Hamster models of cross-modal plasticity. Photomicrographs examples of normal (a), SC lesioned (b) and enucleated (c) hamsters. Column a: top figure showing normal hamster brain with intact superior colliculus and optic chiasm (black arrow heads). At the bottom a simplified schematic representation of the normal visual and auditory pathways. Column b: At the top Superior colliculus (SC) lesioned hamster brain were the SC and optic chiasm are atrophied (black arrow heads). Underneath, diagrams showing the new ectopic retinal projections to the LP in the SC lesioned and to the MG in the SC + ICb lesioned animals. Column c: Enucleated case with an evident SC but complete absence of optic nerves and optic chiasm (arrows). Bottom diagram illustrating the new ectopic auditory projections between the IC and the dLGN to V1. V1, primary visual cortex; V2L, lateral secondary visual cortex; A1, primary auditory cortex; dLGN, dorsal lateral geniculate nucleus; LP, lateral posterior nucleus; MG, medial geniculate nucleus; SC, superior colliculus; IC, inferior colliculus; ICb, inferior colliculus brachium; CN, cochlear nucleus.

In very low-sighted mammals like the blind mole rat (Spalax ehrenbergi), the primary visual thalamic relay, the dorsal lateral geniculate nucleus (dLGN), receives direct atypical subcortical projections from the inferior colliculus (IC) which gets its auditory input from the cochlea [50]. Hence, in these animals, an auditory stimulus can activate neurons in both the dLGN and area V1 [51–53]. Congenitally anophthalmic mutant mice (strain ZRDCT-An) show similar responses. When the dLGN-V1 connectivity is preserved in these mice, there is an increase in the thalamo-cortical projections coming from the lateral posterior (LP) (ancestor of the pulvinar in rodents) and the somatosensory ventroposterior (VP) nuclei [54]. Further, there is development of ectopic innervations of the dLGN and the LP by inputs originating in the dorsal column nuclei (DCN) of the somatosensory system and the IC [55–57]. Chabot et al. found that an auditory stimulation provoked a strong c-fos response in cells of the dLGN and V1 and to a lesser extent in secondary associative visual cortices (V2M and V2L) in these anophthalmic mutants only, compared to C57BL/6 normal controls and enucleated at birth mice that do not develop aberrant projections between IC and the dLGN [46, 56]. More recently, an elegant study using mice mutants that lacked functional rods (Gnat−/−), but had normal cone function, reported that cortical connections of V1 in these animals were similar to those of normal siblings, but there were sparse inputs from the auditory cortex (AC) to area V1. This region also received some abnormal subcortical inputs from the anterior thalamic nuclei, the ventral posterior, the ventral lateral, and the posterior nuclei. While vision generated from a small number of cones appeared to be sufficient to maintain most of the patterns of normal connectivity, the sparse abnormal thalamic inputs to V1, existing inputs from AC, and possibly abnormal inputs to LG and LP may be responsible for generating alterations in the functional organization of V1 of these mice [32]. Taken together, these studies suggest that developmental timing and age at which sensory loss happens are of prime importance to the strength of rewiring and cross-wiring that occurs in cross-modal plasticity. These studies also imply that a prenatal period of spontaneous retinal and/or basic postnatal retinal activities may play a role in shaping differences in sensory reorganization in mammals. This corroborates results obtained in prematurely born animals like hamsters (E15) and opossums (E13,5) in which subcortical reconnections are generally more important. For example, binocular enucleation at birth in these animals also induces strong auditory responses in the primary visual cortex [31, 47, 58, 59]. In enucleated hamsters, single cell electrophysiological recordings have shown that 63% of neurons in the V1 are now responsive to auditory stimulation. This manipulation leads to the formation of direct new ectopic auditory projections from the IC to the dLGN while the connectivity between this nucleus and the visual cortex remains unchanged (Figures 2(a) and 2(c)). These new auditory inputs to the visual thalamus and cortico-cortical connections from A1 are thought to be responsible for the auditory activities found in V1 [47]. Interestingly, reducing or ablating visual thalamo-cortical inputs on the day of birth in normal hamsters significantly increases the number of cortico-cortical projections to V1 arising from both primary nonvisual and associative visual areas in the adult [60]. In enucleated opossums, the cross-modal plasticity and alterations of the subcortical and cortico-cortical afferent circuits are stronger. As a result, in these marsupials, area V1 can now receive ectopic projections from the primary thalamic auditory (medial geniculate nucleus (MG)) and somatosensory (ventrobasal nucleus (VB)) relays as well as new inputs from primary auditory (A1) and somatosensory (S1) cortices. However, no projections between the IC and the dLGN were seen in this model [31]. For comparison purposes, a similar enucleation paradigm in the rat reported a reduction in thalamo-cortical afferents from the dLGN to the visual cortex, a significant increase in the projections from the LP to the V1, and more cortico-thalamic projections between the S1 and the LP in these rats [61]. 

As previously mentioned, it is possible for auditory and/or somatosensory information to reach area V1 in blind mammals via modifications of the cortico-cortical connectivity. Several animal studies in the past decade show the existence of direct anatomical connections between the auditory and visual cortices, more particularly in normal-sighted cats and nonhuman primates [62–66]. Long projections relaying the V1 to other primary sensory cortices are found in several mammalian species such as rats [67], gerbils [68], hamsters [47, 60], and ferrets [69]. Recently, an indirect pathway between the primary auditory and visual cortices through layer V pyramidal neurons in V2L has been identified in the mouse and can be amplified by enucleation at birth. The authors suggest that this A1-V2L-V1 pathway may be involved in multisensory processing and contribute to the auditory activation of the occipital cortex in blind rodents [70]. It is possible that such cortico-cortical connections in normal animals contribute to cross-modal plasticity by being stabilized, reorganized and/or being amplified following any form of sensory loss during development. Taken as a whole, studies so far highlight the importance of putative reorganization of subcortical, thalamo-cortical, and cortico-cortical pathways in the blind brain. 

### 2.2. Artificial Rewiring Paradigms in Hamsters and Ferrets Neonates

**Figure 3:**
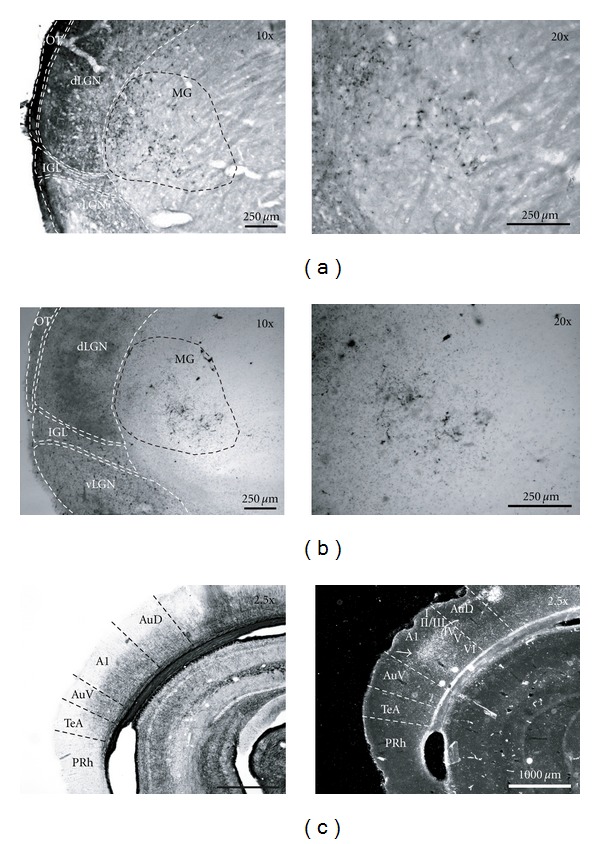
Ectopic retinal projections to the medial geniculate nucleus (MG) in the SC + ICb lesioned (or rewired) hamster. (a) Retinal projections in the MG labelled by intraocular injection of the cholera toxin *β* fragment and (b) co-tagged with wheat germ agglutinin-horse radish peroxidise (WGA-HRP). (c) Gold chloride myelin staining (left panel) and transneuronal labelling, with WGA-HRP (right panel), of new visual thalamo-cortical afferences reaching namely cortical layer IV in the primary auditory cortex (A1) (white arrow head). A1, primary auditory cortex; AuD, dorsal secondary auditory cortex; AuV, ventral secondary auditory cortex; TeA, temporal association cortex; PRh, perirhinal cortex; dLGN, dorsal lateral geniculate nucleus; vLGN, ventral geniculate nucleus; LP, lateral posterior nucleus; MG, medial geniculate nucleus; ot, optic track; IGL, intergeniculate leaflet.

**Figure 4:**
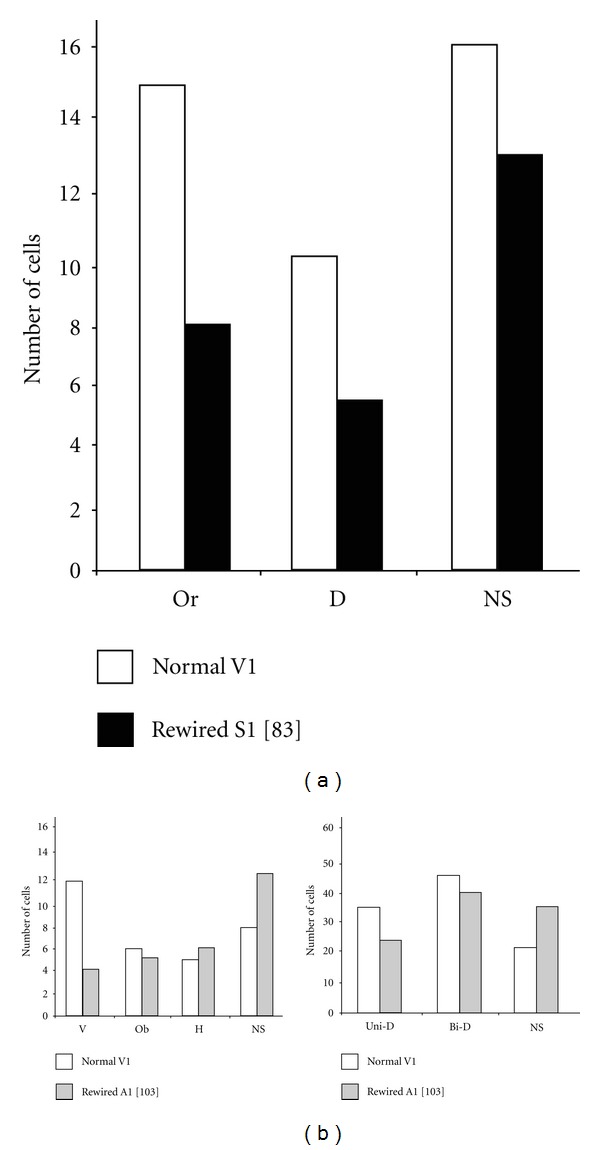
Visual properties of single neurons in auditory and somatosensory cortices of rewired hamsters. These cells that responded to visual stimuli showed orientation selectivity, motion and direction sensitivity with receptive field properties similar with those obtained from neurons in the visual cortex of normal hamsters. (a) Examples of visual responsive neurons in the somatosensory cortex of hamsters with new retinal projections in the somatosensory ventrobasal nucleus (VB) of the thalamus adapted from Metin and Frost [83]. (b) Receptive field properties of visual neurons found in the auditory cortex of hamsters with ectopic retinal terminals in the auditory medial geniculate nucleus (MG). Orientation (left panel) and direction (right panel) selectivity adapted from Frost and collaborators [103]. V, vertical orientation; Ob, oblique orientation; Or, orientation selective; H, horizontal orientation; D, direction selective; Uni-D, unidirectional; Bi-D, bidirectional; NS, non-selective neuron.

**Figure 5:**
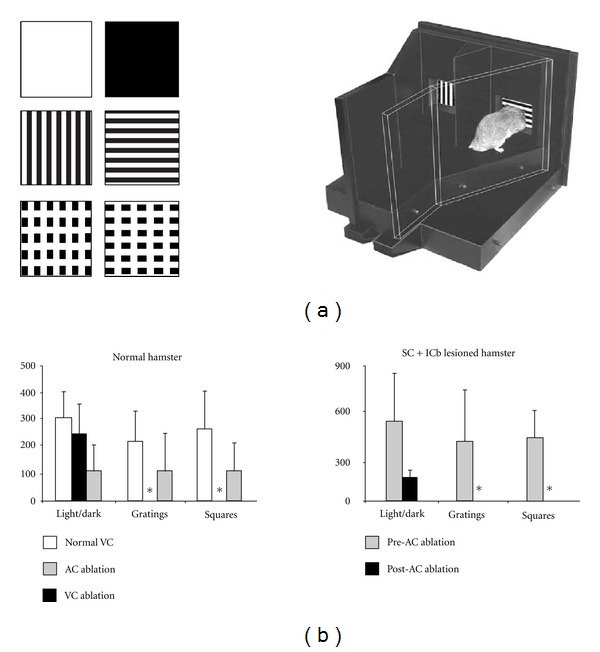
Visually guided behavior in SC + ICb lesioned (or rewired) hamsters. (a) Example of the experimental setup with visual stimuli and Y maze. (b) Histograms showing trials to criterion on the visual discrimination tasks in normal hamsters before and after ablation of visual (VC) and auditory (AC) cortices. (c) Behavior of rewired hamsters before and after AC lesions. At the behavioral level, rewired hamsters can learn visual discrimination tasks as well as normal ones and a lesion of the auditory cortex abolishes this function. In fact, SC + ICb lesioned hamsters with auditory cortex lesions exhibit cortical blindness (*) similar to normal hamsters with visual cortex lesions. These results provide strong evidence for sensory substitution where a given sensory modality acquires the functional properties of a missing one. Adapted from Frost et al. [103] and Ptito et al. [95].

 Cross-modal plasticity changes have also been studied by surgically creating new visual circuits. Schneider pioneered this approach and showed that a lesion of the visual and superficial layers of the superior colliculus (SC) at birth in hamsters (that constantly give birth prematurely at E15) could produce ectopic retinal projections, from surviving ganglionic cells to subcortical sensory relays that normally receive small or no visual inputs [71]. For example, a bilateral lesion of the stratum opticum on postnatal day 1 leads to a fourfold amplification of retinal synapses in the lateral posterior nucleus (LP) of the thalamus, which is a secondary associative visual relay connected to the lateral secondary visual cortex (V2L) in rodents [72, 73] (Figures 2(a) and 2(b)). Frost (in hamsters) and Sur (in ferrets) were the first to optimize this model and demonstrate that, in combination to the superficial SC lesion at birth, surgically cutting the auditory (i.e., the inferior colliculus brachium) or somatosensory (i.e., the medial lemniscus) afferents could lead to the formation of new robust ectopic retinal projections to the auditory medial geniculate nucleus (MG) (Figures 3(a), 3(b)) or to the somatosensory ventrobasal nucleus (VB), respectively [74–80]. At birth thalamo-cortical projections from primary sensory thalamic relays have not yet reached the cortical subplate (this happens at P1 in hamsters and P14 in ferrets). Therefore, by using this experimental paradigm one can alter the nature of sensory activity that reaches the primary auditory or somatosensory cortices during development without changing the original thalamo-cortical connectivity. The new retinal projections inducted in MG or VB are from the three main classical ganglion cell types, form functional synapses, and are retinotopically organized in the host primary auditory (A1 (Figure 3(c))) or somatosensory (S1) cortex, respectively [78–84]. Nevertheless, the molecular and cellular mechanisms involved in the formation of these new ectopic connexions are still unanswered. In the ferret, the morphology of the retinal synapses in the MG are similar to the ones found in the visual CS and dLGN in control animals [85, 86]. Further, retinal afferents conserve their visual organization in the auditory relays [87, 88]. Although the tonotopic organization of thalamo-cortical projections is preserved between MG and A1 in these rewired ferrets [89, 90], it has been shown that the horizontal network in the auditory cortex as well as its contralateral callosal projections is largely modified and is very similar to those normally found in the V1 [91, 92]. *In vivo* electrophysiological recordings of single neurons in both the A1 and S1 indicated that these cells have acquired functional receptive field properties of the visual cortex (i.e., orientation selectivity, motion and direction sensitivity) (see Figure 4) and some also show a bimodal response [83, 93–97]. Using intrinsic signal optical imaging in the A1 of rewired ferrets, visual orientation selectivity columns were found to be similar but broader than those in the V1 of control animals [98–102]. Furthermore, at the behavioral level, these rewired animals can learn visual discrimination tasks and perceive vision with the rewired auditory cortex [95, 103, 104]. Rewired hamsters with no visual cortex can learn visual tasks as well as normal animals, and a lesion of the auditory cortex abolishes this ability and function (Figures 5(a) and 5(b)). In fact, rewired hamsters with auditory cortex lesions exhibit cortical blindness similar to nonrewired hamsters with visual cortex ablations. Overall these results involving intermodal rewiring in neonatal hamsters and ferrets show that sensory information via subcortical thalamic afferents play an important role in shaping anatomical and functional specifications of primary sensory cortices. This suggests that the type of sensory activity and experience can plays an important role in forging parts of the neuroarchitecture of the hosting cortex [1, 4, 105, 106]. 

### 2.3. Can Multisensory Integration Already Be Present in Normal Primary Sensory Cortices?

 The classical modality exclusivity of primary sensory areas has recently been challenged. Observations in a variety of species suggest that each of these domains could already be subjected to influences from other senses in normally reared animals. The first evidence was found in the early 1970s where a study, contested at the time, showed that auditory stimuli could elicit neuronal activity in primary (area 17) and secondary (area 18) visual cortices of normal cats [107]. More recently, transitional multisensory zones of multimodal responsive neurons have been reported at the border of the primary visual cortex in rats [108]. Other recent electrophysiological studies in cats, ferrets, and monkeys have highlighted a low-level influence of other sensory modalities on auditory areas including A1 [69, 109, 110]. These results suggest the possible existence of an important multisensory convergence, occurring at low hierarchical levels, of sensory cortical areas involving feedback, feedforward and lateral cortico-cortical connections and also subcortical inputs. This way a sensory area processing one particular modality could have access, simultaneously, to other unimodal and polymodal sensory information [18, 111]. For example, it is possible that in visually deprived animals or blind humans these putative interactions are modified to permit a greater recruitment of the primary visual cortex by the spared modalities. 

At this point we can assert that cross-modal plasticity that occurs following an early sensory function loss involves important rewiring and cross-wiring processes. However, the question of how thalamo- and cortico- cortical plastic changes, as well as new multisensory integrations, are taking place remains unresolved. A possibly significant mechanism may involve the cortical inhibitory (or GABAergic) interneurons since they are important for visual cortex plasticity and for refinement of sensory information reaching the cortex. 

## 3. The Importance of Cortical GABAergic Interneurons

 The neocortex contains mainly two neuronal types, excitatory (glutamatergic) pyramidal cells and inhibitory nonpyramidal (GABAergic) neurons. These aspiny interneurons are widespread and represent only 15–30% of all neocortical neurons. Inhibitory interneurons include a vast array of subtypes that vary in morphological, physiological, and neurochemical characteristics (e.g., calcium-binding proteins, neuropeptides, ion channels, receptors, and transporters). Further, they target their synapses onto distinct subcellular locations at the postsynaptic level [112–115]. In the cortex, different GABAergic interneuron subtypes were originally classified by expression of calcium-binding proteins parvalbumin (PV), calbindin (CB), or calretinin (CR). Recently, a more accurate classification through expression of several neuropeptides suggests that most inhibitory interneurons in the cortex can be classified in three largely independent populations expressing PV, CB/somatostatin (SS), and CR/vasointestinal peptide (VIP) [112, 113, 115, 116]. Exactly how many specific interneuron subtypes actually exist in the cortices of different species [112, 117–119] is still a matter of debate. Inhibition is critical to a wide range of brain processes specifically in network oscillations and synchronisation, synaptic plasticity, and in preventing runaway excitation. In addition, GABAergic interneurons also regulate nearly all key developmental steps in the cortex, from neuronal proliferation, migration, and differentiation to experience-dependent refinement of local cortical circuits. As a result, disturbances in inhibitory circuits have been involved in a number of neurological disorders, such as epilepsy, schizophrenia, autism, anxiety disorders, and Alzheimer's disease [116, 120, 121]. These GABAergic interneurons are present in all sensory modalities. In sensory cortices interneurons play an essential role in refining sensory receptive fields as well as in confining and modulating sensory afferent activity to sensory cortices. For example, in the visual cortex they shape spacing between cortical columns and strongly influence ocular dominance plasticity [20–22, 122–128].

The large diversity of interneurons suggests that individual inhibitory classes may have unique roles in arbitrating the balance between excitation and inhibition in cortical circuits and plasticity. Development of GABAergic circuits is a prolonged process that begins during midgestation and is complete only by the end of adolescence [114, 116]. PV-positive cells represent the largest subgroup of the GABAergic population in sensory neocortices. Amongst inhibitory neurons in the cortex, the PV subtype is the last to mature in rodents, human and nonhuman primates [129]. The prolonged development of interneurons may constitute a sensitive period where environmental changes can lead to permanent alterations in the inhibitory circuitry. Considering the numerous roles played by GABAergic interneurons in the development, function, and plasticity of cortical networks, combined with their late maturation in the postnatal life, it is reasonable to hypothesize that they could be a key component in gating cross-modal plasticity processes following early sensory deprivation. We will discuss several lines of evidence that stress their possible implication in this context. 

### 3.1. Effects of Sensory Deprivation on the Expression of Calcium-Binding Proteins (CBPs) in Cortical GABAergic Interneurons

**Figure 6:**
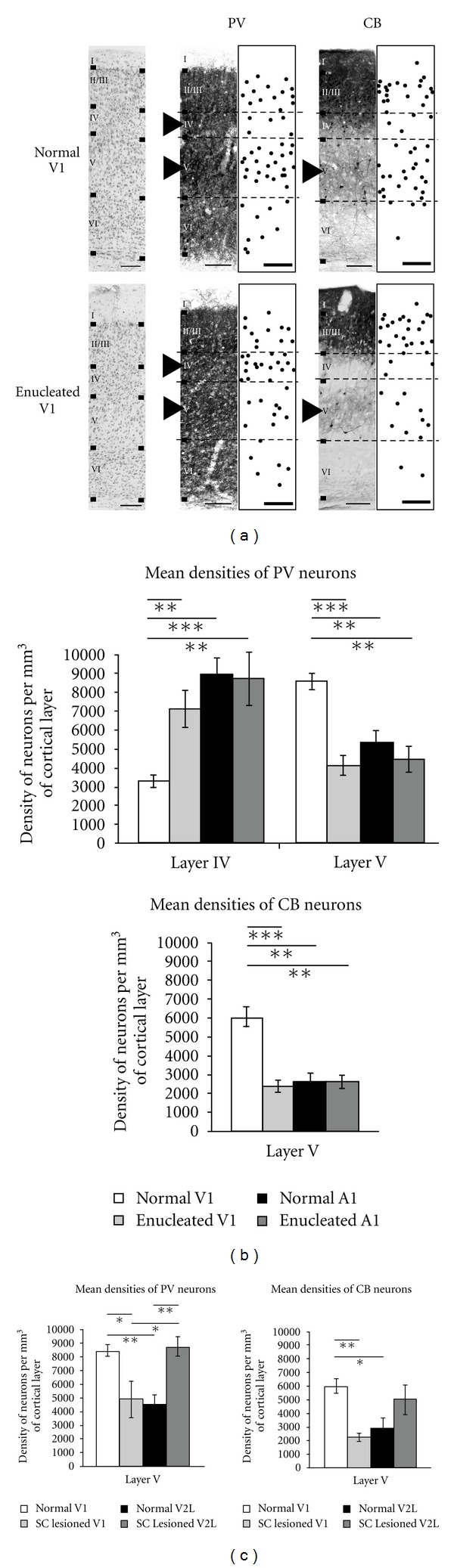
Laminar distribution of PV and CB expressing interneurons in the visual and auditory cortices of normal, enucleated and SC lesioned at birth hamsters. (a) Immunostaining patterns of the two CBP-IR neuronal subpopulations in V1 of normal and enucleated hamster. Left panels: cresyl violet staining with laminar boundaries; middle panels: photomicrographs of the distribution of PV and CB immunoreactivities; right panels: distribution of each CBP-IR neurons (black dots) plotted from three superimposed sampled sections. Black arrows indicate layer IV and V changes for PV-IR and CB-IR neurons between experimental groups. Pial surface of the cortices are at the top. Scale bars 100 *μ*m. (b) Changes in the distributions of PV and CB neurons in layer IV and V of V1 compared to A1 in normal versus enucleated hamsters. (c) Alterations in the distribution of PV and CB interneurons in V1 and V2L of SC lesioned versus normal hamsters. Histograms illustrate the mean density number of neurons per mm^3^ of cortical layer and error bars represent SEM. Significant differences are represented by stars *P < 0.05, **P < 0.01 and ***P < 0.001. Adapted from Desgent et al. [29, 30].

 A large number of studies show that the expression of calcium-binding proteins (CBPs) in cortical GABAergic interneurons can be altered significantly in different sensory areas, olfactory bulb, and in the hippocampus following modifications to the afferent sensory input [130–138]. For example, deafferentation of the primary auditory cortex in ferret at P14 (two weeks before auditory capability) by bilateral ablation of the cochlea causes a reduction in the density of neurons immunoreactive (IR) for GABA, PV, and CB [105]. Ibotenic acid lesions of thalamic afferents from the somatosensory ventroposterior nucleus delay the development of PV and CB interneurons in the rat barrel cortex [139]. More precisely the visual cortex has mostly been studied in rodents, carnivores, and nonhuman primates in that context. Dark rearing, retinal lesions, TTX intraocular injections, mutated loss of photoreceptors in the retinae during development, visual deprivation by eye lid sutures or enucleation have all been shown to modify the expression of the different CBPs (PV, CB, and CR) [140–146]. This was shown for the first time in rats where a monocular enucleation, performed before the critical period at P14, could induce a significant decrease in PV expression in interneurons in the contralateral binocular zone of the primary visual cortex (V1) [142, 147]. The authors had postulated an important role for PV-expressing interneurons in ocular dominance plasticity in this paper. Similar results were also found in the primary visual cortex of adult macaques, wherein following monocular inactivation by intraocular injection of TTX, both PV and CB but not CR were affected in ocular dominance columns associated with the deprived eye. More specifically, in these columns there was significant decrease in the number of neurons, as well as neuropil expressing PV and CB in cortical layers IV to VI and II/III, respectively [141]. Analogous changes were also observed following monocular enucleation or retinal lesions. The main finding was the varying density of perineuronal nets expressing these neurochemical markers between layers II/III and V [140, 143]. In experiments using dark-reared mice, a decrease in the expression of PV but not CB and CR mRNA levels was observed in V1, while monocular deprivation induced no changes [144]. Further studies done in cats show that following dark rearing from birth there is a significant decrease in the total number of cells expressing PV and CR but not CB in areas 17 and 18 [145]. Long-term monocular deprivation in rats decreases the expression of CB but not CR and PV in primary visual cortex, suggesting that the CBPs-IR neuronal subpopulations may be differently affected by the various types of visual deprivation paradigms [148]. More recently, distribution of PV-IR and CB-IR interneurons was studied in the primary visual cortex of CRX−/− mice, where photoreceptors lack outer segments resulting in the complete absence of vision from birth as compared to C57Bl/6 controls [149]. In CRX mutants, there is significant decrease in PV in all layers and of CB only in layers II/III of the primary visual cortex. Developmental results in these mutant mice further suggest that PV expression requires visual activity in V1. In hamsters a lesion of the stratum opticum layer of the SC at birth leads to the death of ~70% of the primary retinal projections to the dLGN-V1 pathway and a fourfold increase in the remaining afferents to the lateral posterior nucleus (LP)-V2L route. Interestingly, in these animals we found changes in the number of PV- and CB- immunoreactive neurons in V1 and V2L as compared to intact hamsters. More precisely, these two populations of neurons were decreased in layer V of V1, but PV interneurons were significantly increased in layer V of V2L in SC-lesioned animals [30] (Figure 6(c)). These results suggest that the decline in visual activity influences PV and CB expressions only in layer V of area V1 whereas the increase of PV-IR cells in layer V of V2L may be correlated with the nurturing presence of new ectopic retinal projections in LP.

Overall, these studies support the idea that different subpopulations of GABAergic neurons are differently influenced by sensory activity depending on their specific anatomical localisation (cortical areas, layers, and modules) and intrinsic cellular properties. It is therefore important to assess the precise function of each specific subtype of interneurons. However, in these experiments, it is not always clear whether the alterations are caused by reduced GABA, PV, CR, and CB immunoreactivity/expression in intact interneurons or by an overall reduction of the GABAergic interneuron population. Furthermore, it is still not clear so far about how the neurochemical identity of a specific interneuron relates to its function. 

### 3.2. GABAergic Networks in Experience-Dependent Plasticity and Critical Period Formation

 Several recent studies in the visual system suggest that an adequate development and function of GABAergic interneurons in the V1 are critical for controlling the onset and time course of critical periods and for the establishment of the cortical circuit architecture that is necessary for the occurrence of ocular dominance (OD) plasticity [128, 150–153]. This was demonstrated for the first time by Hensch et al. where knockout mice, lacking Glutamic Acid Decarboxylase 65 (GAD65, the synaptic isoform of GABA-producing enzyme), showed no closure of the critical period and thus no occurrence of OD plasticity. Nonetheless, this shortage was rescued by cortical infusion of the GABA agonist diazepam (DZ) [126]. Conversely, augmenting inhibitory signaling prematurely launches the critical period making the mice insensitive to monocular deprivation, as is normally the case in the adult mouse [150, 154, 155]. GABA transmission mediated by the *α*1 subunit containing GABA-A receptors has been shown to be mandatory for the induction of the critical period for OD plasticity [155, 156]. In parallel, the precocious development of inhibitory circuitry via exposure to brain derived neurotrophic factor (BDNF) in rodents accelerates the maturation of PV interneurons and triggers early onset of the critical period for visual plasticity [153, 157, 158]. Using kittens and the monocular eye lid suture paradigm, Stryker and collaborators have shown that muscimol-induced blockade in V1 causes inversion of OD plasticity, resulting in a consistent shift in the responsiveness of this cortex in favour of the less-active closed eye [159, 160]. More recently, in similar conditions but using normal sighted cats it was found that infusion of DZ on top of V1 resulted in a widening of ocular dominance column spacing, while reducing inhibition shortened the distance between columns [161]. However, once inhibition is mature, it can restrict cortical plasticity. In the adult visual system of the rat, ocular dominance plasticity is greatly reduced but can be restored to juvenile levels by suppressing inhibition with the antidepressant fluoxetine [162]. Indeed, directly attenuating GABA release by a GAD inhibitor reinstates OD plasticity in adult rats [163]. This is also true for environmental enrichment in old age where diazepam infusion averts the reduction in OD plasticity [164].

More recently, it has been shown by Maffei and collaborators that, in layer IV of binocular V1 in rats, depression of inhibitory synapses on pyramidal neurons is induced when these animals are monocularly deprived for 2 days at the end of the third postnatal week (i.e., before the critical period), whereas potentiation is induced if the monocular deprivation is started in the fourth postnatal week (i.e., within the critical period). During development, these two forms of plasticity shift the balance between circuit excitation and inhibition while the excitatory synaptic drive remains unaffected. Thus, inhibitory plasticity seems to be fundamental in modulating cortical circuit refinement and might be one of the key mechanisms promoting ocular dominance shifts [165]. Such dynamic adjustment of the excitation-inhibition balance may allow the networks to maintain stable levels of activity in the face of variable sensory input. More electrophysiological evidence from this group [148, 166–169] and others [170] suggests that inhibition mediated specifically by fast-spiking (FS) basket-like (PV positive) and regular spiking nonpyramidal (RSNP) (CB or SS positive) interneurons is critically involved in plasticity following deprivation or deafferentation-induced degradation of visual function in V1 of rodents. These effects may however depend upon the age and nature of the visual deprivation and might be differentially regulated across specific cortical layers in the primary visual cortex. Taken as a whole, these studies suggest that maturation of specific subclasses of GABA interneurons is crucial in initiating critical period plasticity, shaping thalamo-cortical afferents, and modulating experience-dependent plasticity in the visual cortex.

### 3.3. Maturation of Inhibitory Networks and Dependence on Sensory Experience

 Although great progress has been made towards understanding both the process of postnatal maturation of excitatory networks and the mechanisms underlying activity-dependent plasticity of excitatory synapses in principal neurons, an understanding of the maturation of inhibitory (GABAergic) circuits has emerged only recently. While the first steps of the development and migration of GABAergic interneurons are likely coordinated by genetic programs, the maturation of these neurons and their synapses are strongly modulated by afferent neuronal activity and experience in both visual and somatosensory cortices. For example, monocular enucleation or dark rearing exposures in rats have been shown to decrease the number of cells and terminals containing GABA and glutamic acid decarboxylase (GAD) [130, 171]. Similar groundbreaking results were also found in the somatosensory cortex, where the unilateral resection of whiskers one day after birth induces a 50% decrease in GABAergic neurons and synapses in layer IV, in the deprived barrels [172]. Conversely and even in the adult brain, mice that undergo excessive stimulation of a single whisker for 24 hours have an increase of inhibitory synapses on dendrites of principal cells in the corresponding barrel [173]. In adult primates, eliminating retinal activity by an intraocular administration of Tetrodotoxin (TTX) reduces the immunoreactivity for GABA, GAD, and the GABA-A receptor in neurons in the areas corresponding to the injected eye in V1 [146, 174–176]. 

More recently, Morales et al. have shown that between the time at which the eyes first open and the end of the critical period for experience-dependent plasticity, the total GABAergic input converging onto pyramidal cells increases threefold in rats. A developmental increase in GABAergic input can be prevented in animals deprived of light since birth, but not in animals deprived of light after a period of normal experience. Thus, sensory experience appears to play a permissive role in the maturation of intracortical GABAergic circuits [177]. In the past decade genetic strategies based on interneuron cell type specific promoters and fluorescent protein reporters have allowed more efficient high-resolution labelling of specific GABAergic interneurons and associated morphology. By using these approaches direct experimental evidence was found linking structural and functional changes of specific inhibitory networks with their sensory experiences *in vivo* and *in vitro*. A significant study in this context from Chattopadhyaya et al. showed that sensory input deprivation using intraocular injections of TTX in mice, and in postnatal organotypic cultures, causes a reduction in the density of perisomatic synapses formed by basket GABAergic neurons in the visual cortex. This sensitivity was restricted to a critical time window during the third postnatal week in mice which correlates with the time course of the critical period for ocular dominance plasticity [178]. These results are consistent with studies done in the barrel cortex in mice that underwent whisker removal from the left mystacial pad at neonatal day 7, until day 15 [134, 138, 179]. These experiments using Glutamate Acid Decarboxylase 67 (GAD67)-Green fluorescent protein (GFP) (delta neo) and wild-type mice showed specific structural anatomical changes, illustrated by a reduction in the number of presynaptic perisomatic inhibitory boutons, specifically from PV interneurons. These changes were associated again with a lack of sensory experience during the second and the third postnatal week. However, the total number of GFP-GAD67 cells (i.e., total number of GABAergic cells) remained unchanged indicating that these changes in PV expression from basket cells appeared to be the major effect of sensory deprivation. Moreover, these modifications were associated with a reduction in the amplitude of evoked intracortical inhibitory synaptic potentials in patch-clamp recordings in deprived versus spared cortices. These results indicate that perisomatic inhibition mediated by PV-positive basket cells was pruned by sensory deprivation. More recently, Jiao and collaborators, using a line of mutant mice that lack activity-dependent BDNF expression (bdnf-KIV), have shown that experience regulates the cortical GABAergic network via activity-driven BDNF expression of principal neurons [138]. Levels of endogenous BDNF protein in the barrel cortex are strongly regulated by sensory inputs from the whiskers. Moreover, the mutant barrel cortex exhibits significantly reduced levels of GABA release only from the PV-expressing fast-spiking (FS) interneurons. Postnatal deprivation of sensory inputs markedly decreases perisomatic inhibition selectively from FS cells in wild-type but not bdnf-KIV mice. These results suggest that postnatal experience, through sensory-driven BDNF expression, controls cortical development by regulating FS cell-mediated perisomatic inhibition *in vivo*. This further highlights that PV (FS) networks can selectively be inhibited by sensory deprivation from the thalamo-cortical afferent pathway.

Together, these results suggest that the properties of local cortical inhibitory network are modified by sensory experience. Thus, postnatal sensory activity is necessary for transformation of immature inhibitory transmission to a mature functional phenotype. Nevertheless, precisely how activity and molecular-driven mechanisms work together to accomplish the remarkable specificity of GABAergic synapse maturation, localization, and formation is not fully understood but is emerging. Several molecular factors have also been implicated in the process such as BDNF, GABA itself, Otx2 homeoprotein, molecular components of the extracellular matrix, and cell adhesion molecules (e.g., chondroitin sulfate proteoglycans (CSPGs), polysialic acid (PSA), and the neural cell adhesion molecule (NCAM)). For example, in mouse visual cortex, PSA is downregulated following eye opening and this decrease has been shown to allow the maturation of GABAergic synapses and the opening, of the critical period for ocular dominance plasticity [180]. For more exhaustive reviews on this topic please refer to the following papers [22, 181–185].

## 4. Alterations of GABAergic Interneurons in Animal Models of Cross-Modal Plasticity

Very few studies have looked at the possible role of GABAergic interneurons in cross-modal plasticity. Alterations in inhibitory circuits were observed qualitatively for the first time in deaf and rewired cross-modal ferrets and concerned modifications in the morphology and proportion of interneurons containing PV and CB. Specifically, CB neurons in A1 of these animals showed an atypical and extended dendritic arborisation in the horizontal axis. However these changes were never studied further with quantitative validation [1, 105]. Interestingly, a recent study done in FVB (GAD-GFP) mice has shown that olfactory deprivation occurring at P12 can lower the number of GABAergic interneurons in the piriform cortex and at the same time increase their number in the barrel cortex, ipsilateral to the lesion, upregulating whisker tactile sensation [28]. This suggests that these neurons are important for cortical sensory compensation and substitution. Recent work carried out in our laboratory, on hamsters enucleated at birth (EH), follows the idea that observed cross-modal plasticity changes may be due to modifications in GABAergic interneurons that express calcium-binding proteins (CBPs) like PV and CB [29, 30]. Since the laminar distribution of these proteins is significantly different in the primary visual and auditory cortices of normal hamsters [186], the induction of aberrant connectivity to these cortices should also be evident at the neurochemical level. Indeed, hamsters enucleated at birth show significant changes in the distribution of CBPs only in their primary visual cortex. Compared to intact hamsters, the density of PV-immunoreactive neurons is higher in layer IV and lower in layer V, whereas the density of CB-immunoreactive cells is significantly lower in layer V of V1 in the enucleated animals (see Figures 6(a) and 6(b)). These results suggest that the affected primary visual cortex may adopt the GABAergic chemical features of the auditory cortex through cross-modal rewiring. 

Several possibilities exist. As described earlier, sensory deprivation generally reduces the expression of PV in primary sensory cortices. Normally, visual activity is an essential requirement in preventing a robust downregulation of PV expression, mainly in cortical layer IV. The functional implications of this decrease, following sensory deprivation in several animal models, have been associated with a reduced inhibition following loss of sight. One can therefore expect a general decrease in PV expression in the V1 of enucleated hamsters, with possible stronger effects in the thalamorecipient cortical layer IV. In CRX−/− mutant mice, there is a significant decrease of PV-IR cells in all layers of the primary visual cortex [149]. This contrasts with the increase in the number of PV-IR cells in layer IV and the decrease observed in layer V of enucleated hamsters. The decrease in PV-IR in mutant mice suggests that parvalbumin expression requires visual activity in V1. There is clearly no visual activity during postnatal development in enucleated hamsters whereas in CRX−/− mice, even with photoreceptors lacking outer segments, there remain waves of spontaneous retinal ganglion cell activity transmitted to the thalamus and cortex before P14. It could therefore be expected that, in enucleated hamsters, one would find an even greater decrease of PV-IR cells in all cortical layers of the V1 as with these mice, but except for layer V this was not the case.

 Significant reduction of expression of both PV and CB in interneurons of layer V of V1 in enucleated hamsters may imply changes in an alternate pathway for cortico-cortical communication between the primary visual cortex and neighbouring-associated areas. Guillery and Sherman proposed that the driving of cortico-cortical projections is mediated by layer V pyramidal neurons that project to the pulvinar of the thalamus (or lateral posterior nucleus (LP) in rodents), which in turn provides the output to higher cortical areas (i.e., the feedforward corticothalamocortical pathway) [187, 188]. Dysfunction of inhibitory interneurons of layer V could play a pivotal role in gating this alternative process and corticocortical communications [26]. It is however unknown as to whether the observed changes in PV and CB expression in interneurons within layer V of EH are directly involved in cortico-cortical networks. In fact, changes in the number of PV-IR and CB-IR neurons in enucleated hamsters might reflect laminar-specific increase and/or decrease of the synaptic drive on these particular neurons in layer IV and V of V1 after enucleation at birth.

Changes in immunoreactive interneurons might reflect two phenomena: (1) a true change in the number/density of PV or CB immunoreactive neurons (e.g., via apoptosis/neurogenesis, impaired migration, suppressed cell proliferation, etc.) or (2) altered immunocytochemical detection levels of the proteins PV or CB, respectively, whose cellular expression might be positively correlated to physiological activity levels [134, 138, 143, 145, 189–191]. Because we did not find any differences in the total population densities for these two proteins, we favour the interpretation that PV and CB expression (changes in synthesis or degradation) in layer IV and V of V1 is altered in EH and that the altered protein levels may be related to the activity of these inhibitory interneurons. However, due to limited knowledge of the physiological function of CBP proteins, the interpretation of the physiological consequences of this early enucleation is complicated. Furthermore, the causal relationship between our anatomical findings and the putative role of these cells in cross-modal plasticity in this animal model remains exploratory.

The observed changes in EH could be explained not only by the absence of postnatal visual input to the V1 but also by the presence of auditory information reaching V1 from new ectopic projections arising from the IC to the dLGN of the thalamus in this rewired model [47]. Another alternative involves cortico-cortical projections originating from the auditory cortex [60]. We hypothesize that these specific changes in the laminar distribution of mostly PV-IR but also CB-IR neurons in V1 could be responsible for shaping auditory response properties of V1 neurons previously observed in enucleated hamsters. The new auditory thalamic afferents into the visual system of the enucleated hamsters could explain the auditory cortex-like distribution pattern of PV-IR neurons in the primary visual cortex. Noteworthy is recent work by Sugiyama et al. which led to the discovery of a novel mechanism explaining how visual input is tied to the onset of ocular dominance plasticity in the visual cortex. This group has shown that a retinal-derived homeoprotein, Otx2, can be directly transferred into V1 through a visual-experience-dependent mechanism. Once Otx 2 has reached the visual cortex, it can nurture specific types of GABAergic interneurons (viz. PV neurons) and modulate critical period plasticity [22, 151, 185, 192, 193]. The study of target genes and proteins of Otx2 could reveal further insights into the machinery linking sensory experience, GABAergic circuit maturation, and plasticity. It is that, as yet unknown homologues of Otx2 might be delivered from other sensory receptor arrays and pathways to precise cortical areas, to promote local inhibitory circuit maturation depending on modality type. In hamsters enucleated at birth, for example, such a molecular factor coming from the auditory system but “redirected” to the primary visual cortex by the cochlea-IC-dLGN pathway could lead to modality-specific changes observed in circuits in absence of visually driven Otx2 but in presence of an auditory homologue. This might explain the altered auditory-like distribution of PV-IR neurons we observed therein [29, 30]. However, the absence of spontaneous electrical activity in the retinal afferents to the lateral geniculate nucleus and the lack of trophic influences of the retina on neurons in the dLGN and from there to area V1 could also be involved in the changes observed in our animal model. It may also be that other sensory modalities, such as somatosensory inputs, could induce the same changes. Injections of HRP into the dorsal column nuclei of adult mice enucleated at birth have shown that ascending somatic sensory axons can be rerouted to the lateral geniculate nucleus [55]. Even if this type of connection has not yet been reported in early enucleated hamsters [47], any combination of these factors, in addition to abnormal auditory rewired inputs to V1 in these animals, could account for the present modifications in the anatomy and laminar circuitry of the V1.

## 5. Future Directions and Conclusion

In conclusion, several animal studies have revealed important properties of inhibitory network alterations in the neocortex following the early loss of a given sensory function. We are just beginning to acquire the necessary knowledge on response properties of GABAergic neurons, their maturation mechanisms, and how they influence sensory and cross-modal plasticity. Hence, we have uncovered only the tip of a very large iceberg in that context. Visual and other sensory cortical circuits are organized at multiple levels of complexity including cortical areas, layers and columns, and specific cell types within these modules. Making sense of the functions of these circuits, from an anatomical point of view, requires linking these circuits to function at each of these levels of complexity. Functional studies on cross-modal plasticity in animals have previously been limited to pharmacological approaches, electrophysiology, tracing or lesion researches that provide poor cell-type specificity and sometimes low-spatial or temporal resolutions. Nowadays, advancements in molecular techniques have made it possible to address questions that were unapproachable just a decade ago. As new methods for single cell two-photon imaging, voltage sensitive dyes for cortical optical imaging, transneuronal viral tracing, *in vivo* MRI spectroscopy (MRS), laser microdissection, transfection and genetic targeting of specific subpopulations of inhibitory interneurons are perfected, we will certainly one day be able to highlight and incorporate better the roles of subtype of GABAergic neurons into multifaceted animal models of cross-modal plasticity. More specifically, one interesting approach concerning this issue would be to use optogenetic in transgenic mouse strains for activation or inhibition of specific GABAergic subpopulations using a viral-mediated transfer of a Cre/loxP transgene controlling the expression of light-activated ion channels in these cells *in vivo* or *in vitro* (e.g., channelrhodopsin ChR and halorhodopsins eNpHR). For example, these would allow us to dissect the function of different neuronal class during awakening behavior or electrophysiology, sensory stimulation, and discrimination tasks with a millisecond resolution. Experiments using these techniques will help us to understand more clearly some important questions in the field: how specific GABAergic subpopulations participate in the rewiring/cross-wiring processes between cortical modalities, what would be the effects of shutting down one population type on cross-modal integration, how these interneurons could integrate inputs from spared modalities, what are the possible multimodal versus unimodal receptive field properties in these cells in normal and sensory deprived cortices, what are the effects on neuronal plasticity as well as neighbouring neuronal networks, are there modality-specific biomarkers that could travel from the sensory receptor periphery to these cortical neurons via a modality experience-dependent-mechanism, and what are the cortical network dynamics between excitatory and inhibitory synapses *in vivo* following the early-life lost of sight? It is going to be a long ride, and experiments to tackle these issues will be very challenging technically. Nevertheless, the clarification of these underlying mechanisms may one day provide clues to develop new therapeutic advances aimed to increase adaptive circuit rewiring following insult to help sensory substitution and recovery.

## References

[1] Pallas SL (2001). Intrinsic and extrinsic factors that shape neocortical specification. *Trends in Neurosciences*.

[2] Majewska AK, Sur M (2006). Plasticity and specificity of cortical processing networks. *Trends in Neurosciences*.

[3] O’Leary DD, Chou SJ, Sahara S (2007). Area patterning of the mammalian cortex. *Neuron*.

[4] Ptito M, Desgent S, Baltes P, Reuter-Lorenz P, Rosler F (2006). Sensory input-based adaptation and brain architecture. *Lifespan Development and the Brain. The Perspective of Biocultural Co-constructivism*.

[5] Krubitzer L (2007). The magnificent compromise: cortical field evolution in mammals. *Neuron*.

[6] Merabet LB, Pascual-Leone A (2010). Neural reorganization following sensory loss: the opportunity of change. *Nature Reviews Neuroscience*.

[7] Ricciardi E, Pietrini P (2011). New light from the dark: what blindness can teach us about brain function. *Current Opinion in Neurology*.

[8] Kupers R, Ptito M (2011). Insights from darkness: what the study of blindness has taught us about brain structure and function. *Progress in Brain Research*.

[9] Ptito M, Kupers R (2005). Cross-modal plasticity in early blindness. *Journal of Integrative Neuroscience*.

[10] Kupers R, Pietrini P, Ricciardi E, Ptito M (2011). The nature of consciousness in the visually deprived brain. *Frontiers in Psychology*.

[11] Noppeney U (2007). The effects of visual deprivation on functional and structural organization of the human brain. *Neuroscience and Biobehavioral Reviews*.

[12] Kupers R, Chebat DR, Madsen KH, Paulson OB, Ptito M (2010). Neural correlates of virtual route recognition in congenital blindness. *Proceedings of the National Academy of Sciences of the United States of America*.

[13] Bubic A, Striem-Amit E, Amedi A, Naumer MJ, Kaiser J (2010). Large-scale brain plasticity following blindness and the use of sensory substitution devices. *Multisensory Object Perception in the Primate Brain*.

[14] Matteau I, Kupers R, Ricciardi E, Pietrini P, Ptito M (2010). Beyond visual, aural and haptic movement perception: hMT+ is activated by electrotactile motion stimulation of the tongue in sighted and in congenitally blind individuals. *Brain Research Bulletin*.

[15] Collignon O, Vandewalle G, Voss P (2011). Functional specialization for auditory-spatial processing in the occipital cortex of congenitally blind humans. *Proceedings of the National Academy of Sciences of the United States of America*.

[16] Kupers R, Beaulieu-Lefebvre M, Schneider FC (2011). Neural correlates of olfactory processing in congenital blindness. *Neuropsychologia*.

[17] Bavelier D, Neville HJ (2002). Cross-modal plasticity: where and how?. *Nature Reviews Neuroscience*.

[18] Driver J, Noesselt T (2008). Multisensory interplay reveals crossmodal influences on “sensory-specific” brain regions, neural responses, and judgments. *Neuron*.

[19] Kadosh RC, Walsh V (2006). Cognitive neuroscience: rewired or crosswired brains?. *Current Biology*.

[20] Sun QQ, Huguenard JR, Prince DA (2006). Barrel cortex microcircuits: thalamocortical feedforward inhibition in spiny stellate cells is mediated by a small number of fast-spiking interneurons. *Journal of Neuroscience*.

[21] Swadlow HA (2003). Fast-spike interneurons and feedforward inhibition in awake sensory neocortex. *Cerebral Cortex*.

[22] Sugiyama S, Di Nardo AA, Aizawa S (2008). Experience-dependent transfer of Otx2 homeoprotein into the visual cortex activates postnatal plasticity. *Cell*.

[23] Gonchar Y, Burkhalter A (1999). Differential subcellular localization of forward and feedback interareal inputs to parvalbumin expressing GABAergic neurons in rat visual cortex. *Journal of Comparative Neurology*.

[24] Johnson RR, Burkhalter A (1996). Microcircuitry of forward and feedback connections within rat visual cortex. *Journal of Comparative Neurology*.

[25] Yamashita A, Valkova K, Gonchar Y, Burkhalter A (2003). Rearrangement of synaptic connections with inhibitory neurons in developing mouse visual cortex. *Journal of Comparative Neurology*.

[26] Callaway EM (2004). Feedforward, feedback and inhibitory connections in primate visual cortex. *Neural Networks*.

[27] Pallas SL, Wenner P, Gonzalez-Islas C (2006). Developmental plasticity of inhibitory circuitry. *Journal of Neuroscience*.

[28] Ni H, Huang L, Chen N (2010). Upregulation of barrel GABAergic neurons is associated with cross-modal plasticity in olfactory deficit. *PLoS ONE*.

[29] Desgent S, Boire D, Ptito M (2010). Altered expression of parvalbumin and calbindin in interneurons within the primary visual cortex of neonatal enucleated hamsters. *Neuroscience*.

[30] Desgent S, Boire D, Ptito M (2008). *Changes in Distribution of Calcium-Binding Proteins in the Visual and Auditory Cortices Following Neonatal Alterations of the Visual System in the Hamster*.

[31] Karlen SJ, Kahn DM, Krubitzer L (2006). Early blindness results in abnormal corticocortical and thalamocortical connections. *Neuroscience*.

[32] Larsen DD, Luu JD, Burns ME, Krubitzer L (2009). What are the effects of severe visual impairment on the cortical organization and connectivity of primary visual cortex?. *Frontiers in Neuroanatomy*.

[33] Rebillard G, Carlier E, Rebillard M, Pujol R (1977). Enhancement of visual responses on the primary auditory cortex of the cat after an early destruction of cochlear receptors. *Brain Research*.

[34] Hunt DL, Yamoah EN, Krubitzer L (2006). Multisensory plasticity in congenitally deaf mice: how are cortical areas functionally specified?. *Neuroscience*.

[35] Jiang H, Lepore F, Ptito M, Guillemot JP (1994). Sensory modality distribution in the anterior ectosylvian cortex (AEC) of cats. *Experimental Brain Research*.

[36] Rauschecker JP, Korte M (1993). Auditory compensation for early blindness in cat cerebral cortex. *Journal of Neuroscience*.

[37] Rauschecker JP (1995). Compensatory plasticity and sensory substitution in the cerebral cortex. *Trends in Neurosciences*.

[38] Rauschecker JP (2002). Cortical map plasticity in animals and humans. *Progress in Brain Research*.

[39] Toldi J, Farkas T, Volgyi B (1994). Neonatal enucleation induces cross-modal changes in the barrel cortex of rat. A behavioural and electrophysiological study. *Neuroscience Letters*.

[40] Toldi J, Feher O, Wolff JR (1996). Neuronal plasticity induced by neonatal monocular (and binocular) enucleation. *Progress in Neurobiology*.

[41] Bronchti G, Schonenberger N, Welker E, Van der Loos H (1992). Barrelfield expansion after neonatal eye removal in mice. *NeuroReport*.

[42] Newton JR, Sikes RW, Skavenski AA (2002). Cross-modal plasticity after monocular enucleation of the adult rabbit. *Experimental Brain Research*.

[43] Rauschecker JP, Tian B, Korte M, Egert U (1992). Crossmodal changes in the somatosensory vibrissa/barrel system of visually deprived animals. *Proceedings of the National Academy of Sciences of the United States of America*.

[44] Yaka R, Yinon U, Rosner M, Wollberg Z (2000). Pathological and experimentally induced blindness induces auditory activity in the cat primary visual cortex. *Experimental Brain Research*.

[45] Yaka R, Yinon U, Wollberg Z (1999). Auditory activation of cortical visual areas in cats after early visual deprivation. *European Journal of Neuroscience*.

[46] Chabot N, Robert S, Tremblay R, Miceli D, Boire D, Bronchti G (2007). Audition differently activates the visual system in neonatally enucleated mice compared with anophthalmic mutants. *European Journal of Neuroscience*.

[47] Izraeli R, Koay G, Lamish M (2002). Cross-modal neuroplasticity in neonatally enucleated hamsters: structure, electrophysiology and behaviour. *European Journal of Neuroscience*.

[48] Kahn DM, Krubitzer L (2002). Massive cross-modal cortical plasticity and the emergence of a new cortical area in developmentally blind mammals. *Proceedings of the National Academy of Sciences of the United States of America*.

[49] Piche M, Chabot N, Bronchti G, Miceli D, Lepore F, Guillemot JP (2007). Auditory responses in the visual cortex of neonatally enucleated rats. *Neuroscience*.

[50] Doron N, Wollberg Z (1994). Cross-modal neuroplasticity in the blind mole rat *Spalax ehrenbergi*: a WGA-HRP tracing study. *NeuroReport*.

[51] Bronchti G, Heil P, Sadka R, Hess A, Scheich H, Wollberg Z (2002). Auditory activation of “visual” cortical areas in the blind mole rat (*Spalax ehrenbergi*). *European Journal of Neuroscience*.

[52] Bronchti G, Heil P, Scheich H, Wollberg Z (1989). Auditory pathway and auditory activation of primary visual targets in the blind mole rat (*Spalax ehrenbergi*): I. 2-deoxyglucose study of subcortical centers. *Journal of Comparative Neurology*.

[53] Heil P, Bronchti G, Wollberg Z, Scheich H (1991). Invasion of visual cortex by the auditory system in the naturally blind mole rat. *NeuroReport*.

[54] Kaiserman-Abramof IR, Graybiel AM, Nauta WJ (1980). The thalamic projection to cortical area 17 in a congenitally anophthalmic mouse strain. *Neuroscience*.

[55] Asanuma C, Stanfield BB (1990). Induction of somatic sensory inputs to the lateral geniculate nucleus in congenitally blind mice and in phenotypically normal mice. *Neuroscience*.

[56] Chabot N, Charbonneau V, Laramée ME, Tremblay R, Boire D, Bronchti G (2008). Subcortical auditory input to the primary visual cortex in anophthalmic mice. *Neuroscience Letters*.

[57] Piche M, Robert S, Miceli D, Bronchti G (2004). Environmental enrichment enhances auditory takeover of the occipital cortex in anophthalmic mice. *European Journal of Neuroscience*.

[58] Kahn DM, Krubitzer L (2002). Retinofugal projections in the short-tailed opossum (*Monodelphis domestica*). *Journal of Comparative Neurology*.

[59] Karlen SJ, Krubitzer L (2009). Effects of bilateral enucleation on the size of visual and nonvisual areas of the brain. *Cerebral Cortex*.

[60] Kingsbury MA, Lettman NA, Finlay BL (2002). Reduction of early thalamic input alters adult corticocortical connectivity. *Developmental Brain Research*.

[61] Negyessy L, Gál V, Farkas T, Toldi J (2000). Cross-modal plasticity of the corticothalamic circuits in rats enucleated on the first postnatal day. *European Journal of Neuroscience*.

[62] Rockland KS, Ojima H (2003). Multisensory convergence in calcarine visual areas in macaque monkey. *International Journal of Psychophysiology*.

[63] Imnocentri GM, Berbel P, Clarke S (1988). Development of projections from auditory to visual areas in the cat. *Journal of Comparative Neurology*.

[64] Clavagnier S, Falchier A, Kennedy H (2004). Long-distance feedback projections to area V1: implications for multisensory integration, spatial awareness, and visual consciousness. *Cognitive, Affective and Behavioral Neuroscience*.

[65] Falchier A, Clavagnier S, Barone P, Kennedy H (2002). Anatomical evidence of multimodal integration in primate striate cortex. *Journal of Neuroscience*.

[66] Hall AJ, Lomber SG (2008). Auditory cortex projections target the peripheral field representation of primary visual cortex. *Experimental Brain Research*.

[67] Vaudano E, Legg CR, Glickstein M (1991). Afferent and efferent connections of temporal association cortex in the rat: a horseradish peroxidase study. *European Journal of Neuroscience*.

[68] Budinger E, Heil P, Hess A, Scheich H (2006). Multisensory processing via early cortical stages: connections of the primary auditory cortical field with other sensory systems. *Neuroscience*.

[69] Bizley JK, Nodal FR, Bajo VM, Nelken I, King AJ (2007). Physiological and anatomical evidence for multisensory interactions in auditory cortex. *Cerebral Cortex*.

[70] Laramee ME, Kurotani T, Rockland KS, Bronchti G, Boire D (2011). Indirect pathway between the primary auditory and visual cortices through layer V pyramidal neurons in V2L in mouse and the effects of bilateral enucleation. *European Journal of Neuroscience*.

[71] Schneider GE (1973). Early lesions of superior colliculus: factors affecting the formation of abnormal retinal projections. *Brain, Behavior and Evolution*.

[72] Crain BJ, Hall WC (1981). The normal organization of the lateral posterior nucleusin the golden hamster and its reorganization after neonatal superior colliculus lesions. *Behavioural Brain Research*.

[73] Ling C, Jhaveri S, Schneider GE (1997). Target- as well as source-derived factors direct the morphogenesis of anomalous retino-thalamic projections. *Journal of Comparative Neurology*.

[74] Sur M, Garraghty PE, Roe AW (1988). Experimentally induced visual projections into auditory thalamus and cortex. *Science*.

[75] Frost DO (1981). Orderly anomalous retinal projections to the medial geniculate, ventrobasal, and lateral posterior nuclei of the hamster. *Journal of Comparative Neurology*.

[76] Frost DO (1982). Anomalous visual connections to somatosensory and auditory systems following brain lesions in early life. *Brain Research*.

[77] Frost DO (1984). Axonal growth and target selection during development: retinal projections to the ventrobasal complex and other “nonvisual” structures in neonatal syrian hamsters. *Journal of Comparative Neurology*.

[78] Frost DO (1986). Development of anomalous retinal projections to nonvisual thalamic nuclei in syrian hamsters: a quantitative study. *Journal of Comparative Neurology*.

[79] Bhide PG, Frost DO (1992). Axon substitution in the reorganization of developing neural connections. *Proceedings of the National Academy of Sciences of the United States of America*.

[80] Bhide PG, Frost DO (1999). Intrinsic determinants of retinal axon collateralization and arborization patterns. *Journal of Comparative Neurology*.

[81] Campbell G, Frost DO (1987). Target-controlled differentiation of axon terminals and synaptic organization. *Proceedings of the National Academy of Sciences of the United States of America*.

[82] Campbell G, Frost DO (1988). Synaptic organization of anomalous retinal projections to the somatosensory and auditory thalamus: target-controlled morphogenesis of axon terminals and synaptic glomeruli. *Journal of Comparative Neurology*.

[83] Metin C, Frost DO (1989). Visual responses of neurons in somatosensory cortex of hamsters with experimentally induced retinal projections to somatosensory thalamus. *Proceedings of the National Academy of Sciences of the United States of America*.

[84] Frost DO (1999). Functional organization of surgically created visual circuits. *Restorative Neurology and Neuroscience*.

[85] Pallas SL, Hahm J, Sur M (1994). Morphology of retinal axons induced to arborize in a novel target, the medial geniculate nucleus. I. Comparison with arbors in normal targets. *Journal of Comparative Neurology*.

[86] Pallas SL, Sur M (1994). Morphology of retinal axon arbors induced to arborize in a novel target, the medial geniculate nucleus. II. Comparison with axons from the inferior colliculus. *Journal of Comparative Neurology*.

[87] Angelucci A, Clascá F, Bricolo E, Cramer KS, Sur M (1997). Experimentally induced retinal projections to the ferret auditory thalamus: development of clustered eye-specific patterns in a novel target. *Journal of Neuroscience*.

[88] Roe AW, Garraghty PE, Esguerra M, Sur M (1993). Experimentally induced visual projections to the auditory thalamus in ferrets: evidence for a W cell pathway. *Journal of Comparative Neurology*.

[89] Pallas SL, Roe AW, Sur M (1990). Visual projections induced into the auditory pathway of ferrets. I. Novel inputs to primary auditory cortex (AI) from the LP/pulvinar comples and the topography of the MGN-AI projection. *Journal of Comparative Neurology*.

[90] Pallas SL, Sur M (1993). Visual projections induced into the auditory pathway of ferrets: II. Corticocortical connections of primary auditory cortex. *Journal of Comparative Neurology*.

[91] Gao WJ, Pallas SL (1999). Cross-modal reorganization of horizontal connectivity in auditory cortex without altering thalamocortical projections. *Journal of Neuroscience*.

[92] Pallas SL, Littman T, Moore DR (1999). Cross-modal reorganization of callosal connectivity without altering thalamocortical projections. *Proceedings of the National Academy of Sciences of the United States of America*.

[93] Frost DO (1990). Sensory processing by novel, experimentally induced cross-modal circuits. *Annals of the New York Academy of Sciences*.

[94] Frost DO, Metin C (1985). Induction of functional retinal projections to the somatosensory system. *Nature*.

[95] Ptito M, Giguère JF, Boire D, Frost DO, Casanova C (2001). When the auditory cortex turns visual. *Progress in Brain Research*.

[96] Roe AW, Pallas SL, Hahm JO, Sur M (1990). A map of visual space induced in primary auditory cortex. *Science*.

[97] Roe AW, Pallas SL, Kwon YH, Sur M (1992). Visual projections routed to the auditory pathway in ferrets: receptive fields of visual neurons in primary auditory cortex. *Journal of Neuroscience*.

[98] Sharma J, Angelucci A, Sur M (2000). Induction of visual orientation modules in auditory cortex. *Nature*.

[99] Sur M, Angelucci A, Sharma J (1999). Rewiring cortex: the role of patterned activity in development and plasticity of neocortical circuits. *Journal of Neurobiology*.

[100] Sur M, Leamey CA (2001). Development and plasticity of cortical areas and networks. *Nature Reviews Neuroscience*.

[101] Horng SH, Sur M (2006). Visual activity and cortical rewiring: activity-dependent plasticity of cortical networks. *Progress in Brain Research*.

[102] Lyckman AW, Sur M (2002). Role of afferent activity in the development of cortical specification. *Results and Problems in Cell Differentiation*.

[103] Frost DO, Boire D, Gingras G, Ptito M (2000). Surgically created neural pathways mediate visual pattern discrimination. *Proceedings of the National Academy of Sciences of the United States of America*.

[104] von Melchner L, Pallas SL, Sur M (2000). Visual behaviour mediated by retinal projections directed to the auditory pathway. *Nature*.

[105] Pallas SL, Schuz A, Miller R (2002). Cross-modal plasticity as a tool for understanding the ontogeny and phylogeny of cerebral cortex. *Cortical Areas: Unity and Diversity*.

[106] Pallas SL, Xu M, Razak KA, Erzurumlu R, Guido W, Molnar Z (2006). Influence of thalamocortical activity on sensory cortical development and plasticity. *Development and Plasticity in Sensory Thalamus and Cortex*.

[107] Fishman MC, Michael CR (1973). Integration of auditory information in the cat’s visual cortex. *Vision Research*.

[108] Wallace MT, Ramachandran R, Stein BE (2004). A revised view of sensory cortical parcellation. *Proceedings of the National Academy of Sciences of the United States of America*.

[109] Brosch M, Selezneva E, Scheich H (2005). Nonauditory events of a behavioral procedure activate auditory cortex of highly trained monkeys. *Journal of Neuroscience*.

[110] Ghazanfar AA, Schroeder CE (2006). Is neocortex essentially multisensory?. *Trends in Cognitive Sciences*.

[111] Kayser C, Logothetis NK (2007). Do early sensory cortices integrate cross-modal information?. *Brain Structure and Function*.

[112] Ascoli GA, Alonso-Nanclares L, Anderson SA (2008). Petilla terminology: nomenclature of features of GABAergic interneurons of the cerebral cortex. *Nature Reviews Neuroscience*.

[113] Markram H, Toledo-Rodriguez M, Wang Y, Gupta A, Silberberg G, Wu C (2004). Interneurons of the neocortical inhibitory system. *Nature Reviews Neuroscience*.

[114] Kilb W Development of the GABAergic system from birth toadolescence.

[115] Druga R (2009). Neocortical inhibitory system. *Folia Biologica*.

[116] Di Cristo G (2007). Development of cortical GABAergic circuits and its implications for neurodevelopmental disorders. *Clinical Genetics*.

[117] DeFelipe J, Elston GN, Fujita I (2002). Neocortical circuits: evolutionary aspects and specificity versus non-specificity of synaptic connections. Remarks, main conclusions and general comments and discussion. *Journal of Neurocytology*.

[118] Hof PR, Glezer II, Nimchinsky EA, Erwin JM (2000). Neurochemical and cellular specializations in the mammalian neocortex reflect phylogenetic relationships: evidence from primates, cetaceans, and artiodactyls. *Brain, Behavior and Evolution*.

[119] DeFelipe J (2002). Cortical interneurons: from Cajal to 2001. *Progress in Brain Research*.

[120] Moult PR (2009). Neuronal glutamate and GABA_A_
receptor function in health and disease. *Biochemical Society Transactions*.

[121] Rossignol E (2011). Genetics and function of neocortical GABAergic interneurons in neurodevelopmental disorders. *Neural Plasticity*.

[122] Porter JT, Johnson CK, Agmon A (2001). Diverse types of interneurons generate thalamus-evoked feedforward inhibition in the mouse barrel cortex. *Journal of Neuroscience*.

[123] Staiger JF, Zilles K, Freund TF (1996). Distribution of GABAergic elements ppstsynaptic to ventroposteromedial thalamic projections in layer IV of rat barrel cortex. *European Journal of Neuroscience*.

[124] Beierlein M, Gibson JR, Connors BW (2003). Two dynamically distinct inhibitory networks in layer 4 of the neocortex. *Journal of Neurophysiology*.

[125] Swadlow HA, Gusev AG (2002). Receptive-field construction in cortical inhibitory interneurons. *Nature Neuroscience*.

[126] Hensch TK, Fagiolini M, Mataga N, Stryker MP, Baekkeskov S, Kash SF (1998). Local GABA circuit control of experience-dependent plasticity in developing visual cortex. *Science*.

[127] Mao R, Schummers J, Knoblich U (2012). Influence of a subtype of inhibitory interneuron on stimulus-specific responses in visual cortex. *Cerebral Cortex*.

[128] Heimel JA, van Versendaal D, Levelt CN (2011). The role of GABAergic inhibition in ocular dominance plasticity. *Neural Plasticity*.

[129] Hensch TK (2005). Critical period plasticity in local cortical circuits. *Nature Reviews Neuroscience*.

[130] Benevento LA, Bakkum BW, Cohen RS (1995). Gamma-aminobutyric acid and somatostatin immunoreactivity in the visual cortex of normal and dark-reared rats. *Brain Research*.

[131] Blasco-Ibanez JM, Martinez-Guijarro FJ, Lopez-Garcia C (1994). Changes in GABA and parvalbumin immunoreactivities in the cerebral cortex of lizards after narine occlusion. *Brain Research*.

[132] Chaudhury S, Nag TC, Wadhwa S (2006). Prenatal acoustic stimulation influences neuronal size and the expression of calcium-binding proteins (calbindin D-28K and parvalbumin) in chick hippocampus. *Journal of Chemical Neuroanatomy*.

[133] Chaudhury S, Nag TC, Wadhwa S (2008). Calbindin D-28K and parvalbumin expression in embryonic chick hippocampus is enhanced by prenatal auditory stimulation. *Brain Research*.

[134] Jiao Y, Zhang C, Yanagawa Y, Sun QQ (2006). Major effects of sensory experiences on the neocortical inhibitory circuits. *Journal of Neuroscience*.

[135] Philpot BD, Lim JH, Brunjes PC (1997). Activity-dependent regulation of calcium-binding proteins in the developing rat olfactory bulb. *Journal of Comparative Neurology*.

[136] de Villers-Sidani E, Simpson KL, Lu YF, Lin RCS, Merzenich MM (2008). Manipulating critical period closure across different sectors of the primary auditory cortex. *Nature Neuroscience*.

[137] Barbado MV, Brión JG, Weruaga E (2002). Changes in immunoreactivity to calcium-binding proteins in the anterior olfactory nucleus of the rat after neonatal olfactory deprivation. *Experimental Neurology*.

[138] Jiao Y, Zhang Z, Zhang C (2011). A key mechanism underlying sensory experience-dependent maturation of neocortical GABAergic circuits in vivo. *Proceedings of the National Academy of Sciences of the United States of America*.

[139] Alcantara S, Soriano E, Ferrer I (1996). Thalamic and basal forebrain afferents modulate the development of parvalbumin and calbindin D28k immunoreactivity in the barrel cortex of the rat. *European Journal of Neuroscience*.

[140] Blumcke I, Weruaga E, Kasas S, Hendrickson AE, Celio MR (1994). Discrete reduction patterns of parvalbumin and calbindin D-28k immunoreactivity in the dorsal lateral geniculate nucleus and the striate cortex of adult macaque monkeys after monocular enucleation. *Visual Neuroscience*.

[141] Carder RK, Leclerc SS, Hendry SH (1996). Regulation of calcium-binding protein immunoreactivity in GABA neurons of macaque primary visual cortex. *Cerebral Cortex*.

[142] Cellerino A, Siciliano R, Domenici L, Maffei L (1992). Parvalbumin immunoreactivity: a reliable marker for the effects of monocular deprivation in the rat visual cortex. *Neuroscience*.

[143] Botelho EP, Guimarães Martins Soares J, da Silva Pereira S, Fiorani M, Gattass R (2006). Distribution of calbindin-28kD and parvalbumin in V1 in normal adult *Cebus apella* monkeys and in monkeys with retinal lesions. *Brain Research*.

[144] Tropea D, Kreiman G, Lyckman A (2006). Gene expression changes and molecular pathways mediating activity-dependent plasticity in visual cortex. *Nature Neuroscience*.

[145] Sanchez-Vives MV, Nowak LG, Descalzo VF, Garcia-Velasco JV, Gallego R, Berbel P (2006). Crossmodal audio-visual interactions in the primary visual cortex of the visually deprived cat: a physiological and anatomical study. *Progress in Brain Research*.

[146] Jones EG (1993). GABAergic neurons and their role in cortical plasticity in primates. *Cerebral Cortex*.

[147] Berardi N, Domenici L, Parisi V, Pizzorusso T, Cellerino A, Maffei L (1993). Monocular deprivation effects in the rat visual cortex and lateral geniculate nucleus are prevented by nerve growth factor (NGF): I. Visual cortex. *Proceedings of the Royal Society B*.

[148] Mainardi M, Landi S, Berardi N, Maffei L, Pizzorusso T (2009). Reduced responsiveness to long-term monocular deprivation of parvalbumin neurons assessed by c-Fos staining in rat visual cortex. *PLoS ONE*.

[149] Goldshmit Y, Galley S, Foo D, Sernagor E, Bourne JA (2010). Anatomical changes in the primary visual cortex of the congenitally blind Crx- / - mouse. *Neuroscience*.

[150] Fagiolini M, Hensch TK (2000). Inhibitory threshold for critical-period activation in primary visual cortex. *Nature*.

[151] Hensch TK (2005). Critical period mechanisms in developing visual cortex. *Current Topics in Developmental Biology*.

[152] Hensch TK, Fagiolini M (2005). Excitatory-inhibitory balance and critical period plasticity in developing visual cortex. *Progress in Brain Research*.

[153] Huang ZJ, Kirkwood A, Pizzorusso T (1999). BDNF regulates the maturation of inhibition and the critical period of plasticity in mouse visual cortex. *Cell*.

[154] Iwai Y, Fagiolini M, Obata K, Hensch TK (2003). Rapid critical period induction by tonic inhibition in visual cortex. *Journal of Neuroscience*.

[155] Fagiolini M, Fritschy JM, Löw K, Möhler H, Rudolph U, Hensch TK (2004). Specific GABA_A_
circuits for visual cortical plasticity. *Science*.

[156] Katagiri H, Fagiolini M, Hensch TK (2007). Optimization of somatic inhibition at critical period onset in mouse visual cortex. *Neuron*.

[157] Hanover JL, Huang ZJ, Tonegawa S, Stryker MP (1999). Brain-derived neurotrophic factor overexpression induces precocious critical period in mouse visual cortex. *The Journal of Neuroscience*.

[158] Gianfranceschi L, Siciliano R, Walls J (2003). Visual cortex is rescued from the effects of dark rearing by overexpression of BDNF. *Proceedings of the National Academy of Sciences of the United States of America*.

[159] Hata Y, Tsumoto T, Stryker MP (1999). Selective pruning of more active afferents when cat visual cortex is pharmacologically inhibited. *Neuron*.

[160] Reiter HO, Stryker MP (1988). Neural plasticity without postsynaptic action potentials: less-active inputs become dominant when kitten visual cortical cells are pharmacologically inhibited. *Proceedings of the National Academy of Sciences of the United States of America*.

[161] Hensch TK, Stryker MP (2004). Columnar architecture sculpted by GABA circuits developing cat visual cortex. *Science*.

[162] Vetencourt JFM, Vetencourt M, Sale A (2008). The antidepressant fluoxetine restores plasticity in the adult visual cortex. *Science*.

[163] Harauzov A, Spolidoro M, DiCristo G (2010). Reducing intracortical inhibition in the adult visual cortex promotes ocular dominance plasticity. *Journal of Neuroscience*.

[164] Sale A, Maya Vetencourt JF, Medini P (2007). Environmental enrichment in adulthood promotes amblyopia recovery through a reduction of intracortical inhibition. *Nature Neuroscience*.

[165] Maffei A, Lambo ME, Turrigiano GG (2010). Critical period for inhibitory plasticity in rodent binocular V1. *Journal of Neuroscience*.

[166] Maffei A, Turrigiano GG (2008). Multiple modes of network homeostasis in visual cortical layer 2/3. *Journal of Neuroscience*.

[167] Maffei A, Turrigiano G (2008). The age of plasticity: developmental regulation of synaptic plasticity in neocortical microcircuits. *Progress in Brain Research*.

[168] Maffei A, Nataraj K, Nelson SB, Turrigiano GG (2006). Potentiation of cortical inhibition by visual deprivation. *Nature*.

[169] Maffei A, Nelson SB, Turrigiano GG (2004). Selective reconfiguration of layer 4 visual cortical circuitry by visual deprivation. *Nature Neuroscience*.

[170] Lazarus MS, Huang ZJ (2011). Distinct maturation profiles of perisomatic and dendritic targeting GABAergic interneurons in the mouse primary visual cortex during the critical period of ocular dominance plasticity. *Journal of Neurophysiology*.

[171] Ribak CE, Robertson RT (1986). Effects of neonatal monocular enucleation on the number of GAD-positive puncta in rat visual cortex. *Experimental Brain Research*.

[172] Micheva KD, Beaulieu C (1997). Development and plasticity of the inhibitory neocortical circuitry with an emphasis on the rodent barrel field cortex: a review. *Canadian Journal of Physiology and Pharmacology*.

[173] Knott GW, Quairiaux C, Genoud C, Welker E (2002). Formation of dendritic spines with GABAergic synapses induced by whisker stimulation in adult mice. *Neuron*.

[174] Hendry SH, Jones EG (1986). Reduction in numbers of immunostained GABAergic neurones in deprived-eye dominance columns of monkey area 17. *Nature*.

[175] Hendry SH, Jones EG (1988). Activity-dependent regulation of GABA expression in the visual cortex of adult monkeys. *Neuron*.

[176] Hendry SH, Miller KL (1996). Selective expression and rapid regulation of GABA_A_
receptor subunits in geniculocortical neurons of macaque dorsal lateral geniculate nucleus. *Visual Neuroscience*.

[177] Morales B, Choi SY, Kirkwood A (2002). Dark rearing alters the development of GABAergic transmission in visual cortex. *Journal of Neuroscience*.

[178] Chattopadhyaya B, Di Cristo G, Higashiyama H (2004). Experience and activity-dependent maturation of perisomatic GABAergic innervation in primary visual cortex during a postnatal critical period. *Journal of Neuroscience*.

[179] Sun QQ (2007). The missing piece in the “use it or lose it” puzzle: is inhibition regulated by activity or does it act on its own accord?. *Reviews in the Neurosciences*.

[180] Di Cristo G, Chattopadhyaya B, Kuhlman SJ (2007). Activity-dependent PSA expression regulates inhibitory maturation and onset of critical period plasticity. *Nature Neuroscience*.

[181] Chattopadhyaya B (2011). Molecular mechanisms underlying activity-dependent GABAergic synapse development and plasticity and its implications for neurodevelopmental disorders. *Neural Plasticity*.

[182] Huang ZJ (2009). Activity-dependent development of inhibitory synapses and innervation pattern: role of GABA signalling and beyond. *Journal of Physiology*.

[183] Huang ZJ, Di Cristo G, Ango F (2007). Development of GABA innervation in the cerebral and cerebellar cortices. *Nature Reviews Neuroscience*.

[184] Pizzorusso T, Medini P, Landi S, Baldini S, Berardi N, Maffei L (2006). Structural and functional recovery from early monocular deprivation in adult rats. *Proceedings of the National Academy of Sciences of the United States of America*.

[185] Sugiyama S, Prochiantz A, Hensch TK (2009). From brain formation to plasticity: insights on Otx2 homeoprotein. *Development Growth and Differentiation*.

[186] Desgent S, Boire D, Ptito M (2005). Distribution of calcium binding proteins in visual and auditory cortices of hamsters. *Experimental Brain Research*.

[187] Guillery RW (1995). Anatomical evidence concerning the role of the thalamus in corticocortical communication: a brief review. *Journal of Anatomy*.

[188] Guillery RW, Sherman SM (2002). Thalamic relay functions and their role in corticocortical communication: generalizations from the visual system. *Neuron*.

[189] Caillard O, Moreno H, Schwaller B, Llano I, Celio MR, Marty A (2000). Role of the calcium-binding protein parvalbumin in short-term synaptic plasticity. *Proceedings of the National Academy of Sciences of the United States of America*.

[190] Patz S, Grabert J, Gorba T, Wirth MJ, Wahle P (2004). Parvalbumin expression in visual cortical interneurons depends on neuronal activity and TrkB ligands during an early period of postnatal development. *Cerebral Cortex*.

[191] Vreugdenhil M, Jefferys JGR, Celio MR, Schwaller B (2003). Parvalbumin-deficiency facilitates repetitive IPSCs and gamma oscillations in the hippocampus. *Journal of Neurophysiology*.

[192] Hensch TK (2005). Recovery in the blink of an eye. *Neuron*.

[193] Huang ZJ, Di Cristo G (2008). Time to change: retina sends a messenger to promote plasticity in visual cortex. *Neuron*.

